# The Mucous Membrane of the Mouth

**Published:** 1886-06

**Authors:** Edwin A. Cormack


AMERICAN JOURNAL

OF

DENTAL SCIENCE.

VOL. XX. THIRD SERIES.—JUNE, 1886. NO. 2.

ARTICLE I.

### THE MUCOUS MEMBRANE OF THE MOUTH.

*With special reference to its Physiology and Pathology, and the part played by its Secretions in the production of Dental Caries.*

BY EDWIN A. CORMACK, L. R. C. P. & S., L. D. S. EDIN.

The subject of the mucous membrane of the mouth is an important one, the whole significance of which has been greatly underrated by the dental profession till within the last few years. In the opinion of many eminent observers its secretions are important factors in the causation of dental caries.

To understand the rationale of their operation we must possess a correct knowledge of the connections of the mucous membrane with other parts, its functions and the agencies which its secretions are subjected to in the mouth. In order to fulfil this object systematically we will discuss in succession its anatomy, physiology, and pathological anatomy. The special diseases of the gum I have reserved for discussion at a future period.

Having referred to the anatomy of the mucous membrane and the gum, of the PHYSIOLOGY the author says:— The mucous membrane yields a secretion—mucus. The mucus is derived from certain of the epithelial cells which elaborate it within their protoplasm and then extrude it.

Lymphoid elements also escape from the mucosa, and passing through the epithelial cells, reach the surface and form the swollen spherules known as mucus corpuscles.

Mucus is a colorless viscous fluid. It contains the debris of shed epithelium, mucus corpuscles, and mucin.

Mucus corpuscles are nucleated masses of protoplasm, similar in size to white blood corpuscles, but containing, unlike the latter, one or more nuclei. Even at the normal temperature, they exhibit amæboid movements. These cells are probably modified epithelial cells of the columnar variety. Mucin is an albuminoid substance that occasions the viscosity of mucus fluids; it thereby facilitates the passage of food over mucous surfaces. It is not coagulated by heat, but is precipitated by alcohol and acetic acid. The precipitate is not soluble in excess of the acid. The precipitate swells up in water, but is not dissolved in it; it is however, readily soluble in alkalies.

Mucin is largely produced in the mucous glands, and in the secreting cells of the salivary glands, especially in the sub-maxillary and sub-lingual. Mucin, in composition, differs only from albumin in containing no sulphur. Albuminoids contains C. H. O. N.; like proteids, they yield leucin and tyrosin, one or both, when subjected to hydrolytic treatment. Albuminoids readily decompose into a number of simpler bodies.

GENERAL PATHOLOGY OF MUCOUS MEMBRANE.

The diseases of the mucous membrane of the mouth are generally of an inflammatory character, and are most common in childhood, resulting usually from intestinal disorders, bad hygiene, and want of local cleanliness.

Inflammations may also be induced by noxious substances acting on the external surface of the membrane. The intensity, extent, and duration of the inflammations vary greatly; and a number of different forms are distinguished accordingly.

Inflammations may be divided into acute and chronic, and, according to their character, into catarrhal, croupous diphtheritic, and gangrenous.

Hyperæmia is very commonly met with in the mouth—where it is of long standing, it may be referred to the atonic condition of the walls of the blood-vessels from malnutrition. The mucous membrane becomes intensely reddened, and, at the same time, the secretion is increased.

In catarrhal inflammations, the characteristic feature is a morbid increase of the secretion of the membrane. The catarrhal secretion is furnished partly by the blood-vessels and partly by the epithelial cells. There is an exudation from the capillaries of liquor sanguinis, containing numerous white blood corpuscles. This exudation is always mingled with secretions from the epithelial cells. These cells normally produce mucus from their protoplasmic contents, the columnar cells chiefly; in catarrh this is much increased. Great quantities of glassy mucus are thus deposited on the membrane along with the normal secretion of the mucus glands.

The inflammation may now subside, and the membrane recover. But should any further irritation arise, other changes will take place. The epithelial cells begin to be shed, and secretion is rendered turbid by their presence, then we have what is termed epithelial catarrh.

In the later stages, there is an excessive desquamation of the epithelium and an abundant extravasation of the white leucocytes. Many forms of catarrhal inflammation are thus characterized by an almost purulent secretion, and are described as purulent catarrh.

Catarrhal inflammations are usually transient; sometimes, however, the process becomes chronic.

In addition to the alterations in the secretion and the epithelial cells, there is an infiltration of cells into the mucosa, and sometimes into the sub-mucosa. Where the epithelium has been lost by desquamation, repair is effected by multiplication of the remaining epithelial cells.

The inflammation may, however, become intensified, so that the tissues perish over some considerable extent. When the infiltration is extreme, the tissue perishes by necrosis. In this way ulcers are produced. The lymph follicles are often the seat of inflammation and ulceration. Ulcers starting in them are known as follicular ulcers. When catarrh passes into ulceration, the inflammatory exudation usually extends far beyond the limits of the ulcer.

When the inflammation is of long duration, a certain amount of fibrous hyperplasia takes place. The openings of the glands often become obstructed, and they are thus distended into cysts.

### INFLAMMATORY AFFECTIONS OF THE MOUTH.

The slightest degree is known as *Erythema*. It is characterized by redness, sense of heat, and sometimes considerable tenderness, but is not usually attended with acute pain. This may rapidly disappear or pass into the more severe form known as

*Catarrhal Stomatitis*. In this form the surface is intensely red, the secretion of the membrane is increased, and the epithelium desquamates. Over the surface of the gums, lips, and cheeks, the redness and swelling are generally uniform, but in the hard palate they may appear in streaks and patches.

When the inflammatory exudation is abundant, clear vesicles are sometimes found on the tongue, lips and cheeks, where the epithelial covering is thicker than elsewhere, and prevents the free escape of the exuded liquid. As the vesicles break, small ulcers, covered with a whitish film of detritus, may be formed in their place. The mucus glands become swollen, giving rise to greyish or greyish-red elevations surrounded by a reddened areola. When the duct becomes obstructed with mucoid cells, the gland may be dilated into a tiny cyst by the retention of its secretion.

The catarrhal secretion contains at first few cells, but later the proportion becomes increased. The cells are in part extravasated leucocytes, in part desquamated epithelial cells. If the latter remain on the surface they may accumulate so as to form a whitish or discolored grey and brown deposit or fur.

When the gums are involved they swell and rise up between the teeth, around the necks of which they ulcerate. In some cases this ulceration does not cease until it has extended into the alveoli and destroyed altogether the connections of the teeth, which become loosened and fall out.

Catarrhal stomatitis is generally the result of some mechanical or chemical irritation of the mucous membrane; when the irritation is local, like that caused by a carious tooth, the stomatitis is likewise local. There are many diseases which set up imflammation of the mouth. In measles a muscular eruption appears, in scarlet fever a punctate, or diffuse scarlet eruption. In small-pox, chicken-pox, pemphigus, and in foot-and-mouth disease there are eruptions of vesicles and pustules, which pass through the same stages as those of the skin.

Simple catarrhal inflammation frequently occurs during the period of dentition, when it is often accompanied by fever.

*Erysipelatous Inflammation* may extend from the skin to the mouth, or may begin in the mouth, caused by direct action of irritant bodies, as by scalding drinks, acrid or corrosive substances taken in the mouth. It is characterized by livid redness and much swelling, and sometimes even vesiculation. The tongue is the part most affected.

*Apthous Stomatitis* is distinguished by the appearance on the catarrhal mucous membrane of small whitish or slightly yellowish patches (apthæ) from the size of a millet seed to that of a pea.

They are surrounded by a livid border and may coalesce into larger patches. These apthæ consist of a solid exudation lying between the fibrous tissue and the epithelium—(Bohn). The exudation may be re-absorbed and the apthæ then disappear, or the epithelial covering is broken through, the fibrinous film exposed, and gradually separated by the growth of epithelium advancing beneath it from the margin.

As the epithelium is reproduced simultanously with the separation of the fibrin, no ulcers are in general produced. The eruption occurs in successive crops, and may thus be kept up for weeks.

It occurs chiefly in children who are teething. It also occurs in connection with sore throat, pneumonia, gastric catarrh, the acute exanthemata, diphtheria, ague, whooping-cough.

This affection has no connection with any invasion of fungi.

*Ulcerative Stomatitis* is an affection which always starts from the alveolar margin of the gums—(Bohn). It begins with redness, swelling, and loosening of the gums around the teeth. The alveolar border becomes rounded and swollen, with blunt proceses rising up between the teeth. Hæmorrhage is not uncommon at this stage. In the second stage the margin of the swollen gum becomes discolored, and the tissue softens and breaks down into a yellowish friable mass—ulcers are thus formed, which rapidly deepen. The ulcerative process may extend directly to the contiguous parts of the cheek and gums, and may work downwards till it attacks the periosteum of the bony structures, leading to necrosis.

The affection is usually acute—children are especially liable to it. It attacks people who are badly nourished or debilitated by disease, such as scrofulous disorders, intestinal complaints, typhoid, diabetes or scurvy; damp, cold, and impure air favor its appearance.

Local irritations may lead to it, as in cases of chronic poisoning by mercury, phosphorous, lead, and copper. The form, which is due to long continued phosphorous poisoning, is apt to extend deeply into the tissues, and so give rise to periostitis and necrosis of the bones.

*Noma or cancrum oris* is an allied but more severe disease. It may begin as an ulcerative disease, or appear independently. In the former, the disintegration of the tissue of the gum extends rapidly, and the tissue breaks down into pulpy gangrenous mass.

In the latter, the first symptoms is the appearance of a livid swelling on the inner surface of the cheek, near the angle of the mouth, accompanied by the free flow of foul saliva; a patch of greyish yellow infiltration then appears, and this speedily breaks down and becomes gangrenous.

As the disease progresses, a purplish spot appears on the outside of the cheek, this becomes black, and gangrene sets in and spreads. It is generally confined to one side. Once the gangrene has commenced, it may spread to a considerable extent, advancing very rapidly: noma is generally fatal. It attacks poor and debilitated children, usually between the ages of two and twelve.

Suppurative inflammation of the mucous membrane of the mouth and partly underlying it should be distinguished from ulcerative stomatitis and noma. It may affect any part but appears most commonly in the tongue and gums. In the latter, it frequently arises in connection with carious teeth. The gum becomes red and swollen, and, presently, pus forms beneath the surface. This is termed a gumboil.

*Croupous Stomatitis.*—When the mucous membrane is so injured that its epithelium is destroyed here and there, and its blood-vessels so much injured as to allow of free exudation of their contents, coagulation of the latter may take place. This forms a false membrane, consisting of fibrinous filaments and granules, beset with pus cells or of shining homogeneous blocks representing cells that have undergone coagulative necrosis.

The false membrane is connected with the underlying structures by fibrinous filaments; but can be readily pulled off, showing the reddened surface of the mucous membrane beneath. To produce coagulation, the exudation must be of an inflammatory nature and the cells necrotic.

*Diphtheritic Stomatitis.*—When a mucous membrane is injured in such a way that its epithelium dies without desquamation, while its blood-vessels are damaged and pour out an abundant exudation, it sometimes happens that the dead epithelial cells become saturated with the exuded liquid, and then pass into a peculiar condition of rigidity resembling coagulation.

The seat of this change appears as a dull grayish raised patch, surrounded by red and swollen mucous membrane. The exudation, is rich in albumen, and the cells take the form of a kind of course network. The sub-epithelial areolar tissue is beset with filaments of fibrin and leucocytes. Hæmorrhages are not uncommon.

Inflammations of this kind, in which the tissue itself coagulates into a solid mass, are called diphtheritic: and when the necrosis and coagulation extend only to the epithelium the affection is called superficial diphtheritis. Inasmuch, then, as the croupous membrane consists essentially of coagulated exudation, croupous inflammation is at once to be distinguished from superficial diphtheritis, in which the epithelium coagulates *en masse.*

Deep or parenchymatous diphtheritis is characterized by the coagulation not merely of the epithelium but also of the underlying connective tissue.

Wedl states that this affection gives rise to a general inflammation of the root membranes over the whole row of teeth in the upper or lower jaws.

"Croupous inflammation of the gum is an affection of considerable importance, both on account of its sudden occurrence and also because it is liable to assume a diphtheritic character."

"In the first stage the edge of the gum is covered with a whitish-grey membraniform exudation. . . The mucous membrane deprived of its epithelium is slightly swollen, tender to the touch, and bleeds easily." Generally the exudation appears upon the margin of the lower jaw which faces towards the lips and cheeks, whence it spreads gradually over the whole anterior and posterior edges of the gums. The exudation degenerates very rapidly into an offensive, sanious mass.—(Wedl.)

### PARASITIC AFFECTIONS.

The oral cavity is always infested by a multitude of vegetable micro-parasites which gain entrance to it from without, and find in it a fitting soil for their growth.

Moulds, yeasts, and bacteriæ are all met with ; of the latter micro-cocci and sarcinæ occur, as wells as bacilli and spirilla. Where cleanliness is not observed, they may occasionally set up putrefactive decomposition. Measles, scarlatina, small-pox, diphtheria, &c., all give rise to inflammatory conditions of the mouth ; and as we regard these diseases as due to micro-parasites, we must assume that the corresponding pathogenous organisms gain access to the tissues of the mouth.

Saccharomycis albicans—muguet, or thrush fungus—is a special fungus of the mouth, generally referred to as Oidium albicans. It is one of the yeasts, and is therefore akin to the mycoderma vini (mother of vinegar) or saccharomyces cerevisiæ. The scum which forms on the surface of alcoholic liquors, and leads to their transformation into vinegar, contains this yeast fungus—the mother of vinegar. Yeast cells not only set up fermentation directly, but they yield an unorganized ferment which changes cane sugar into grape sugar.

They have no power of invading living tissue and are of little pathological importance, and it is only under specially favorable conditions they are able to grow freely. There is usually no great supply of fermentable saccharine matter available for them. The presence of acids does not check their development. Although probably always present in the mouth, the sub-epithelial tissues are only invaded when by antecedent changes, constitutional or other, the resisting power of the tissues has been considerably diminished. As it occurs in the mouth, it assumes the form of rounded or oval cells, seldom filaments. It gives rise to minute, whitish, slightly raised specks on the mucous membrane. They are generally to be found on the inner surface of lips and over the tongue. As they grow they coalesce into whitish films. After a time the film is cast off, the surface beneath appearing red and sometimes eroded. The fungus grows mainly in the middle layers of the stratified epithelium. From this position the fungus may penetrate into the deeper layers and reach the fibrous structure. Its progress downwards is marked by inflammation.

Thrush is a disease which manifests itself in very young children, and in adults debilitated by exhausting diseases. Its growth is favored by the use of cows' milk or starchy food, and by imperfect cleansing of the infant's mouth.

### HYPERTROPHY AND ATROPHY.

The epithelium of the mouth is constantly being shed and continually renewed by regenerative multiplication. In catarrhal affections this shedding is a prominent feature, and deposits are formed on the surface of the mucous membrane. This deposit is added to by the remains of food and by rapidly growing fungus parasites. In this way a continuous film or fur is produced.

Hyperplasia of the connective tissue is due either to some chronic inflammatory cause or to congenital conditions.

Inflammatory hyperplasia is most commonly met with in connection with the gums. It gives rise to circumscribed tumor-like thickenings.

Atrophy of the gums and of the alveolar parts of the jaws is apt to follow upon the loss of the teeth, and is normally present in old age.

The results, then, of our investigations into diseases of the mucous membrane show that they are for the most part of an inflammable character, caused in some cases by direct action of irritants taken into the mouth, in others by disorders acting initially at more distant parts of the alimentary canal. We have also seen that the characteristic eruptions of the exanthemata make their appearance in the mouth; that a weak habit of body predisposes, and that malnutrition and bad hygiene are important factors in their causation. Parasites also contribute their evil influences.

Inflammation, if slight, increases the secretions; if more severe, it impairs the vitality of the mucous membrane by desquamation of its epithelium and interruption of its functions. It may destroy the epithelial covering, and even the underlying tissues, by infiltration, ulceration, or necrosis.

By infiltration or by hyperplasia it may produce obstruction of the gland ducts, and consequent cysts; or, where the processes are excessive, total obliteration.

### INFLUENCE OF THE SECRETIONS OF THE MUCOUS MEMBRANE ON THE PRODUCTION OF CARIES.

From the earliest times the causation of caries has occupied the attention of those who devoted themselves to dental pathology. Since the time of Vespasian (90—120 B. C.), when Aretæus confessed that "the cause of toothache is known only to God," many investigators, more enterprising than he, have made innumerable observations and propounded theories more or less satisfactory. It was known about the beginning of this century that certain acids found in the mouth were capable of decomposing the teeth; but caries was generally believed to be due to some inflammatory agency situated within the tooth, hence the term *caries interna.*

In 1835, Robertson of Birmingham considered that caries was due to "the corrosive chemical action of the solid particles of food which had been retained, and have undergone a process of putrefaction or fermentation in the several parts of the teeth best adapted for their reception."

Since then numerous theories have been advanced. Mr. Tomes, in 1859, advocated the chemico-vital theory. He believed that acids, whose origin he referred to the mucous and saliva, resolved the tooth into its histological elements, and that the dentine exerted a resistive force, or alternatively, that caries was to some extent inflammatory.

Magitot, in 1869, elaborated the theory of resistance and consolidation of dentine, but eliminated the idea of inflammation.

The year after, Leber and Rottenstein demonstrated the presence of leptothrix granules in the dentinal tubes. Hence they denied the reaction of dentine.. To caries of the enamel they ascribe a chemical cause.

In 1870 Wedl verified part of Leber and Rottenstein's experiments, but considered acid essential to the disease. He believed caries to be due to abnormal secretions of the oral membranes and salivary glands; and that in consequence of their fermentations, acids were formed which acted on the teeth.

In 1873 Tomes abandoned all idea of vital action as a part of caries, and regarded the calcification of the fibrils as doubtful.

In 1881 Underwood and Milles believed the disease to be due to micro-organisms; also that previous experiments showing caries to be due to acids were void, because septic conditions prevailed.

In 1882, Miller, of Berlin, demonstrated that micro-organisms do not precede decalcification, but that they are the cause of the disintegrations of the matrix after the removal of the lime salts by acids.

Mr. Mossman, of Iowa City—from whose articles on Dental Caries, published lately in the *Dental Cosmos*, most of the above account of the history of the different theories has been taken—sums up as follows :—

The vital or inflammatory theory of caries, an inflammatory disease having a central origin.

The chemical theory recognized the existence of acids in the mouth and their capacity to dissolve the lime salts.

The chemico-vital theory is a combination of the two above mentioned. According to this belief the acids act upon the inorganic tissues; the irritation is conveyed to the pulp, which reacts against the invasion of the disease by throwing out lime salts into the fibrils.

The parasitic theory. The enamel being removed by acids, the organisms penetrate the tubuli of the dentine and proliferate there, according to some writers, merely expanding the tubuli for the better penetration of acids, and, according to others, generating themselves an acid by their action upon the organic matter.

The chemico-parasitic theory. This gives to acids the first place, to their agency all decalcification is due. Following this process come the micro-organisms, which penetrate only when decalcification is sufficient to permit their advance. Then they are found in great numbers, and cause the putrefaction of the organic mass.

The chemico-putrefective theory is the same as the above, save that the micro-organisms are only incidental to the putrefactive process, and have no part in the disease.

Messrs. Underwood and Milles describe the microscopical appearance of carious dentine as follows:—

"The tubes are filled with micro-organisms. They appear to penetrate the canals at first in single file, and then accumulate in vast numbers to encroach upon the matrix. Here and there a narrow line of bacteria or micrococci penetrate beyond the sphere of visible decay. Besides the disintegrated tissues and foreign particles, there is found abundance of leptothrix buccalis.

"The micro-organisms consists of micrococci, rod-shaped and oval bacteria, and short bacilli.

"The number of sections of carious teeth cut and examined is now so enormous, that observers feel justified in assuming that the presence of micro-organisms is indispensable to the process"—(Sewill). Acids are necessary for the primary decalcifications.

The sources of these acids may be referred to the processes of fermentation and putrefaction, which are continually going on in the mouth.

Fermentation requires the presence of a ferment, which may be either organized, that is, living, or unorganized. Living ferments grow and multiply at the expense of the matters in which they occur. Yeast may be cited as an example of the former, and ptyalin of the latter. Moulds, yeasts, and bacteria, are all met with in the mouth. They gain access to it from without (Ziegler).

The mucous secretion and the secretion from the salivary glands combine to form mixed saliva. All starchy or saccharine substances taken into the mouth are converted into grape sugar by ptyalin.

Grape sugar, when acted upon by a yeast, the torula, undergoes alcoholic fermentation ; the presence of the bacterium aceti induces acetous fermentation. Stomatitis and caries of the teeth have been observed to be especially frequent in the case of chronic drunkards, and it is probable that beer drinkers suffer more from these affections than those who indulge in liquors possessing a larger percentage of alcohol. May not the acetous fermentation of the liquor imbibed cause these conditions ? Another yeast, the mycoderma vini, transforms the alcoholic products of the first torula fermentation into acetic acid.*

Milk is taken as food, acted upon by the bacterium lactis, lactic acid is produced.

Putrefaction also requires the presence of a ferment, and we know that the bacterium termo is that ferment—(Ziegler). Naegleli, Pasteur, Lister, and others regard putrefactive fermentation as the direct result of the vegetation of the bacteria. Bacteria, as they grow and multiply, withdraw from the nutrient liquid the elements they require

---

*Naegleli maintains that the torula and the myco-derma are not distinct species, and, according to Grawitz, the white patches, known as thrush, are due to the presence of the myco-derma, the mycelial filaments and spores of which are distinguished as belonging to the Oidium Albicans. (Ziegler).

for building up their cells. These are chiefly C. H. O. N. from carbohydrates and albuminoids. The necessary inorganic compounds are derived from salts containing sulphur, phosphorous, magnesium, and potassium. None of the bacteria can develop without water—(Ziegler).

When albuminoids undergo decomposition, we have formed formic, *acetic*, butyric, valerianic, caproic, and *lactic* acids; combined with ammonia, or other organic alkalies, leucin and tyrosin, sulphuretted hydrogen, carbolic acid, and various other substances, When proteids are entirely decomposed, there remains a substance rich in fats, in earthy and ammoniacal salts, phosphates, and nitrates. (Rutherford).

In hyperæmia of the mucous membrane, there is an excessive secretion of mucus. The chief constituent of mucus is mucin, an albuminoid substance, ready splitting into a number of simpler bodies. Animal substances are taken as food into the mouth. These are principally composed of albuminoids and fats. One of the products of their decomposition is lactose, which breaks up into lactic acid.

We may say, then, that putrefactive fermentation may be set up by the action of bacteria on the mucous secretion, either alone or along with remains of food. When the mucus is extremely viscid, it may determine the location of caries by causing the adhesion of food, &c., to particular surfaces.

In the two processes of fermentation and putrefaction, two acids—acetic and lactic—are formed. These both act powerfully on the teeth.

According to Leber and Rottenstein's experiments, a solution of acetic acid of the strength of 1 in 1000 decalci fied in 17 days the enamel and adjacent dentine of a tooth placed in it; a 10 per cent. solution of lactic acid showed during the same time, a very decided action on another tooth. It is probable, then, that acetic and lactic acids, along with other acids, produced in the putrefactive decom position of albuminoids, dissolve the lime-salts of the dental tissues.

Miller, of Berlin, states that micro-organisms are never to be found in any but decalcified tissues of the tooth, and this is probable, as bacteria cannot live without water, and it is difficult to believe that they can go on in advance without it.* We may, therefore, conclude that they await decalcification. When the dentine is reached, channels are hollowed out to admit the fluid collecting in the cavity behind. In this float the organisms, deriving nourishment partly from it and partly from the disintegration of the organic matrix, by this means producing, and setting free, the acids of putrefactive fermentation to continue the process indefinitely.

The exclusion of water, as a means of stopping the invasion of caries, has long been recognized, and the energies of successive generations of dentists have been devoted to the application of water-tight fillings.

We may conclude, then, that the secretion of the mucous membrane takes part in certain fermentative and putrefactive processes, when in sufficient quantity, either alone or in combination with saliva or foods. That micro-organisms superinduce putrefactive decompositions of albumin-

---

*Drs. Leber and Rottenstein, who, in 1877-78, published their researches on the origin of caries through the action of leptothrix buccalis, differ from Miller.

There is, they say, no manner of doubt that the elementary parts of the fungus penetrate into the interior of the canals, and there develop in a manner to acquire a relatively considerable diameter.

It also results from the very fact of the dilatation of the canals, that the appearance of the fungus is not accidental, and, that it is not by a purely passive action that it invaded the canals. It is necessary that there be a proliferation of spores infinitely minute and innumerable of the fungus to effect the dilatation of the tubules. It is, moreover, very important that the elements of the leptothrix should have, at a certain stage, a mobility of their own, in virtue of which they easily penetrate the interior canals. As for the rest, we have met in the canals with only the granular masses of the leptothrix, and never the filaments which appear to show themselves only at the surface. [The granular masses are probably only the disintegrated tissues.—E. A. C.]

oids; that such processes produce acids which act by decalcifying the dental tissues, and so make way for the microorganisms found by Miller and others. That these organisms, by removing certain constituents of the organic matrix of the dentine, produce putrefaction, and consequent disintegration. That the locality of caries may be determined by the collection of organic matter on dental surfaces, coated with viscous mucous.

The section on pathology has been largely taken from Ziegler's Pathological Anatomy. I have also referred to the following works:—Salter—Dental Pathology and Surgery. Harris—Principles and Practice of Dentistry. Wedl—The Pathology of the Teeth. Tomes—Dental Surgery. Tomes—Dental Anatomy.—*Odonto-Chirurgical Society of Scotland.*—*London Dental Record.*

